# Spatial Reconfiguration of Living Stems and Snags Reveals Stand Structural Simplification During Moso Bamboo (*Phyllostachys edulis* (Carrière) J.Houz.) Invasion into Coniferbroad-Leaf Forests

**DOI:** 10.3390/plants14111698

**Published:** 2025-06-02

**Authors:** Xi Chen, Xiumei Zhou, Songheng Jin, Shangbin Bai

**Affiliations:** Jiyang College, Zhejiang A&F University, Zhuji 311800, China; 20189014@zafu.edu.cn (X.C.); zhouxm0324@163.com (X.Z.); shjin@zafu.edu.cn (S.J.)

**Keywords:** bamboo invasion, point pattern analysis, quantitative characteristics, tree mortality, community dynamics

## Abstract

In subtropical regions of China, the expansion of Moso bamboo has become increasingly prominent, resulting in massive mortality of original trees in adjacent forest stands. Significant changes have also occurred in the population characteristics and spatial distribution patterns of these native tree species. This study aims to examine the impacts of Moso bamboo (*Phyllostachys edulis*) expansion on the successional dynamics of coniferous and broad-leaved mixed forests. Three sample plots were successively set up in the transition zone from bamboo to conifer and broad-leaved forest, including conifer and broad-leaved mixed forest (CF), transition forest (TF), and Moso bamboo forest (MF); a total of 72 10 m × 10 m quadrats (24 per forest type) were included. The species composition, diameter class structure and distribution pattern of living stems and snags (dead standing stems) were studied. The results showed that during the late expansion phase of bamboo, the density of living stems and snags separately increased by 2234 stems·ha^−1^ and 433 stems·ha^−1^, basal area increments of 23.45 m^2^·ha^−1^ and 7.81 m^2^·ha^−1^. The individuals with large diameter in living stems and snags gradually decreased, and the distribution range of the diameter steps mainly narrowed to 10–15 cm. On the scale of 0–10 m, the spatial pattern of standing stems changed from random and weak aggregation distribution to strong aggregation distribution and then to weak aggregation and random distribution in the three stands, while the overall distribution of snags in the three stands was random. The spatial correlation between living stems and snags evolved from uncorrelated in CF, to significant positive correlation in TF, and then to positive correlation and uncorrelation in MF. These results indicated that the bamboo expansion accelerated the mortality rate of the original tree species, leading to the diversity of tree species decreased, the composition of diameter classes was simplified, the degree of stem aggregation increased, and intra- and inter-species competition became the main reasons for tree death.

## 1. Introduction

The growth, development, and survival of individual trees are determined not only by their intrinsic characteristics but also by their capacity to exploit environmental resources and their interactions with neighboring trees. Such interactions critically shape tree growth, morphology, and survival [1,2]. The combined effects of stand succession and regeneration processes, as well as underlying ecological mechanisms, lead to tree mortality across all developmental stages [3]. Consequently, the species composition, diameter class structure, and spatial arrangement of living stems and snags (standing dead stems) can reflect the current growth status and successional stage of a forest stand.

Moso bamboo (*Phyllostachys edulis*), native to subtropical China, occupies approximately 69.78% of the total bamboo forest area in China, with its distribution exhibiting a sustained rapid increase [4]. As a clonal plant, this species typically colonizes adjacent habitats through underground rhizomes. It demonstrates rapid growth, achieving vertical dominance in the canopy layer within 2–3 months after shoot emergence [5]. Under natural low-light conditions beneath forest canopies, it is generally hypothesized that native species cannot invade neighboring forests without anthropogenic disturbances. However, forest ecosystems may exhibit limited resistance when confronted by shade-tolerant invaders [6]. Although not classified as invasive in China, Moso bamboo possesses vigorous rhizomatous expansion capacity and may autonomously encroach on surrounding natural forests through patch-based colonization [7]. Notably, bamboo shoot elongation is primarily dependent on carbohydrate reserves from clonal root systems [8], rendering this process largely independent of light availability [9]. Studies have shown that Moso bamboo is continuously invading adjacent plant communities (with an average expansion rate of 1.36 m/y) [10] and suppresses the growth of other vegetation through allelopathic effects [11], resulting in a series of ecological and environmental issues such as simplified forest community structure [12], reduced species diversity [13], impeded natural regeneration [14], and decreased soil organic carbon content [15]. During the expansion of Moso bamboo into adjacent forests, snags (dead standing stems with height more than 1.8 m)—a legacy of living stem succession—are frequently observed. As critical components of forest ecosystems, snags significantly influence carbon balance [16], nutrient cycling [17], and forest regeneration [18], while providing essential habitats for diverse organisms [19]. The species composition and spatial patterns of snags are modulated by stand characteristics (e.g., community composition, habitat heterogeneity), stem diameter classes, and disturbance regimes, exhibiting distinct spatial heterogeneity [20]. Therefore, a comprehensive, multi-scale investigation of the species assemblages and spatial configurations of both living stems and snags during bamboo encroachment is imperative to elucidate: (1) mechanisms underlying stem mortality, (2) successional trajectories of forest communities, and (3) material-energetic dynamics within ecosystems.

Spatial distribution patterns represent one of the core issues in ecological research [21], serving as the foundation for understanding underlying ecological processes. As a fundamental attribute of populations, their spatial distribution emerges from the integrated effects of multiple ecological processes, reflecting not only interspecific and intraspecific relationships within communities but also population–habitat interactions [22]. Given the scale-dependent nature of spatial distribution patterns, point pattern analysis can be employed to treat individuals as spatial points based on their coordinate data, thereby quantifying multiscale spatial patterns and elucidating mechanisms driving forest community assembly [23].

Given the strong competitive capacity of Moso bamboo, research attention on the spatial structure of bamboo forests has increased in recent years [24,25,26]. However, only a limited number of studies have addressed the impacts of bamboo expansion on the spatial distribution patterns of adjacent plant populations [14,27]. And the impacts of bamboo encroachment on spatial configurations of living stems and snags within forests remain poorly understood. In China’s subtropical region, coniferous and broad-leaved mixed forests—transitional ecosystems between pure coniferous and evergreen broad-leaved forests—exhibit high stability, species richness, structural complexity, and functional diversity. These forests play critical ecological roles in maintaining biodiversity and forest community heterogeneity. Currently, Moso bamboo encroachment has become increasingly pronounced in these ecosystems [13]. During bamboo expansion into surrounding forests, dynamic shifts in species composition, diameter class distributions, and spatial patterns of both living stems and snags inevitably exert profound impacts on forest ecosystem stability and developmental trajectories.

To address these knowledge gaps, this study aims to achieve the following objectives: (1) elucidate mechanisms driving tree mortality during bamboo encroachment; (2) quantify population spatiotemporal patterns in bamboo-forest ecotones; (3) advance bamboo expansion ecology; and (4) provide a scientific basis for conserving mixed forests and regulating bamboo expansion.

## 2. Results

### 2.1. Effects of Moso Bamboo Expansion on Composition of Living Stems and Snags

With the expansion of Moso bamboo, the density and basal area of both standing living stems and snags within the forest have significantly increased. Species diversity declines, and Moso bamboo becomes the absolute dominant plant. As presented in Table 1, TF and MF exhibited markedly elevated densities and basal areas for both living and dead standing stems compared to the CF. During the late expansion phase (MF), living stem density increased by 2234 stems·ha^−1^ and snags by 433 stems·ha^−1^ relative to the pre-expansion phase (CF), with respective basal area increments of 23.45 m^2^·ha^−1^ and 7.81 m^2^·ha^−1^.

At the species level (Table 1), the pre-expansion coniferous–broadleaf forest (CF) was dominated by living trees of *Schima superba*, *Pinus massoniana*, and *Cunninghamia lanceolata*, which exhibited relatively high proportions of snags. Notably, dead *Cunninghamia lanceolata* stems constituted 27.6% of its living counterparts, representing the highest mortality ratio among all species. In contrast, dominant species (*Castanopsis eyrei*, *Liquidambar formosana*, and *Cyclobalanopsis glauca*) displayed significantly lower dead-to-living stem ratios. In the intermediate expansion phase (TF), Moso bamboo demonstrated pronounced dominance in both stem density (relative abundance: 76.3%) and basal area (relative dominance: 50.0%) among living stems. Non-bamboo species collectively showed no significant variation in these metrics compared to pre-expansion levels, except for a significant increase in the density of *Liquidambar formosana*. The snags were predominantly composed of *Phyllostachys edulis* (relative abundance: 38.0%), *Cyclobalanopsis glauca* (relative abundance: 20.4%), *Pinus massoniana* (relative abundance: 12.0%), *Castanopsis eyrei* (relative abundance: 12.0%), and *Schima superba* (relative abundance: 12.0%). Compared with CF, the stem density of snags in TF increased significantly; among them, *Phyllostachys edulis* had the highest relative abundance of snags (38.0%), *Pinus massoniana* had the highest relative significance (48.3%), and *Cyclobalanopsis glauca* had the highest ratio of snags to living stems (50.0%). During the late expansion stage of Moso bamboo (MF), Moso bamboo (basal area: 34.03 m^2^·ha^−1^) dominated both in quantity and relative dominance among living stems. Among other species, *Castanopsis eyrei* (basal area: 2.74 m^2^·ha^−1^), *Pinus massoniana* (basal area: 2.34 m^2^·ha^−1^), and *Cyclobalanopsis glauca* (basal area: 1.02 m^2^·ha^−1^) showed no significant differences in either individual count or basal area at breast height compared to the pre-expansion stage (CF). In contrast, *Schima superba* (basal area: 0.08 m^2^·ha^−1^) exhibited a notable reduction in both living stem density and basal area compared to CF levels. *Cunninghamia lanceolata* and *Liquidambar formosana*, along with other minor species, had nearly disappeared from the community. Regarding snags, Moso bamboo maintained its absolute dominance in both abundance and relative significance. No statistically significant differences were observed in either snag numbers or basal area measurements of other tree species between MF and CF stages.

### 2.2. Effects of Moso Bamboo Expansion on Diameter Class Structure of Living Stems and Snags

**Figure 1:**
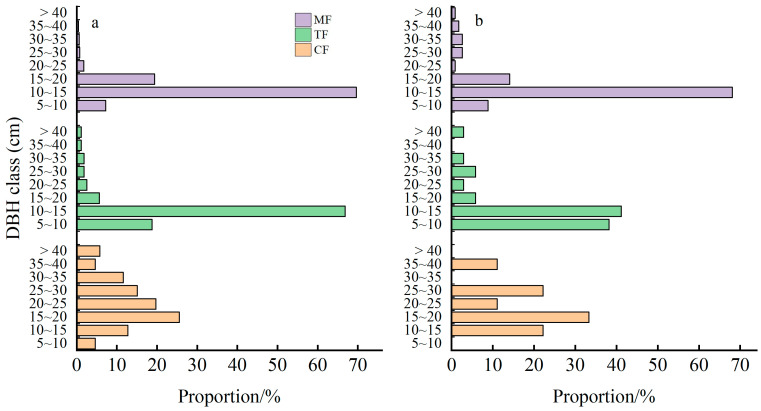
Changes in diameter class of living stems (**a**) and snags (**b**) during the Moso bamboo expansion into conifer and broad-leaved mixed forests.

As shown in Figure 1, the diameter class structure of living stems in the three forest stands generally exhibited unimodal distributions, with the proportional distribution initially increasing and then decreasing as diameter classes advanced. In the CF, diameter classes were predominantly distributed in 10–15, 15–20, 20–25, 25–30, and 30–35 cm, cumulatively accounting for 84.9% of the total. The TF showed concentration in 5–10 cm and 10–15 cm classes, representing 18.8% and 66.9% of the total, respectively. In the MF, diameter classes were primarily distributed in 10–15 cm and 15–20 cm, constituting 69.7% and 19.4% of the total. These results indicate that as Moso bamboo expanded into the CF, large-diameter living stems progressively declined, and the range of diameter class distribution narrowed. Furthermore, as revealed in Table 1, the diameter classes of Moso bamboo within MF exhibited an increasing trend compared to those in TF.

The diameter class structures of snags and living stems were significantly aligned across the three forest stands. With Moso bamboo expansion, the proportional representation of large-diameter snags decreased markedly, while that of small-diameter classes increased significantly. Notably, in TF, the dominance of small-diameter snags was particularly pronounced, with their individual counts overwhelmingly prevailing in the diameter class distribution.

### 2.3. Effects of Moso Bamboo Expansion on Spatial Distribution Patterns of Living Stems and Snags

**Figure 2:**
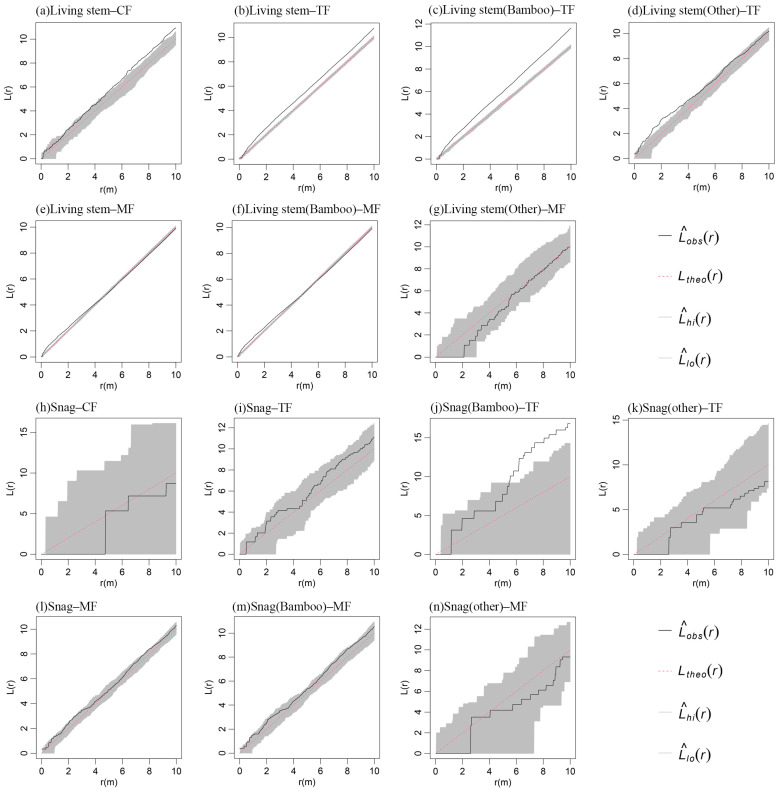
Point pattern analysis of living stems (**a**–**g**) and snags (**h**–**n**) during the Moso bamboo expansion into conifer and broad-leaved mixed forests.

**Figure 3:**
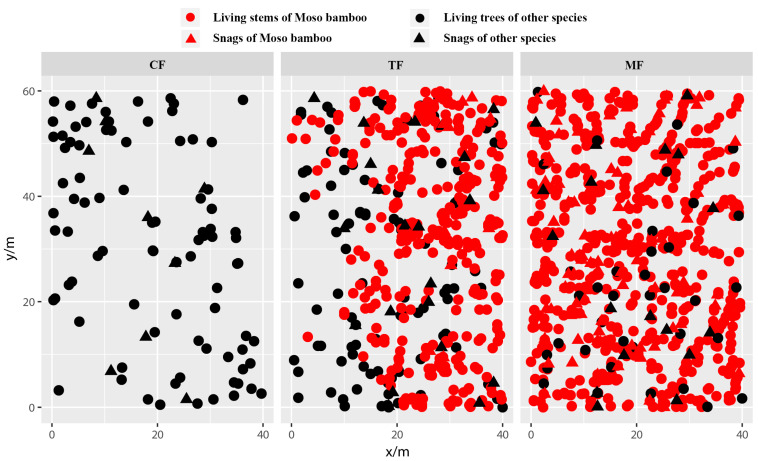
Spatial distribution of living stems and snags during the Moso bamboo expansion into conifer and broad-leaved mixed forests.

During the pre-expansion stage of Moso bamboo, living stems in CF exhibited random distribution at the 0–5 m scale, transitioning to aggregated distribution with increasing spatial scale (Figure 2a). In the middle expansion stage, living stems in TF demonstrated aggregated distribution across 0–10 m scales (Figure 2b). Specifically, Moso bamboo showed aggregated distribution at 0–10 m scales with intensifying aggregation as scale increased (Figure 2c), while other tree species displayed aggregated distribution at 0–4 m scales that shifted to random distribution with expanding scale (Figure 2d). During the late expansion stage, living stems in MF exhibited aggregated distribution at 0–4 m scales, gradually transitioning to random distribution with increasing spatial scale (Figure 2e). Since over 90% of the living stems in the MF were Moso bamboo, the spatial distribution pattern of living stems in the MF closely mirrored that of the Moso bamboo population itself (Figure 2e,f). In contrast, other tree species exhibited random distribution across 0–10 m scales (Figure 2g). This observation indicates significant changes in the spatial distribution of living stems across the three stand types (CF, TF, and MF) during different stages of bamboo expansion (Figure 2a–g), which aligned with the visual patterns observed in the spatial distribution maps (Figure 3).

Snags in the three stand types primarily exhibited random distribution across 0–10 m scales (Figure 2h–n), aligning with the visual patterns observed in the spatial distribution maps (Figure 3). An exception occurred in the bamboo–tree mixed forest, where dead Moso bamboo plants displayed aggregated distribution at 6–10 m scales (Figure 2j).

### 2.4. Spatial Associations of Standing Stems Across Moso Bamboo Expansion Stages

**Figure 4:**
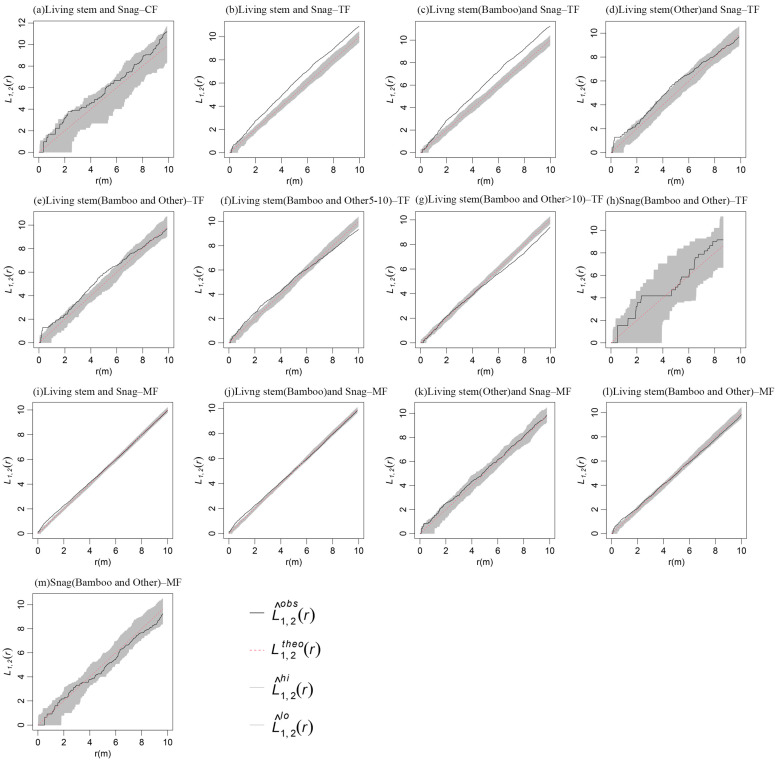
Spatial association of standing stems and snags during the Moso bamboo expansion into conifer and broad-leaved forests.

Spatial correlation analysis of living stems and snags across the three stand types, as well as between Moso bamboo and other tree species during the middle and late expansion stages, revealed that during the pre-expansion stage of Moso bamboo, no significant spatial association was observed between living stems and snags in the CF at 0–10 m scales (Figure 4a).

During the middle expansion stage of Moso bamboo, living stems and snags in TF exhibited significant positive spatial correlations (Figure 4b). Notably, the positive correlation between living Moso bamboo and snags intensified (Figure 4c), while living stems of other species showed no significant correlation with snags across most spatial scales (Figure 4d). Living Moso bamboo and living stems of other species displayed significant or near-significant positive correlations at 0–6 m scales, but these associations became non-significant beyond 6 m (Figure 4e). Specifically, Moso bamboo and saplings of other tree species (5 ≤ DBH < 10 cm) demonstrated significant or near-significant positive correlations at 0–4 m scales, gradually shifting to negative correlations with increasing spatial scale (Figure 4f). In contrast, Moso bamboo exhibited no significant correlation with large-diameter stems (DBH > 10 cm) of other species at 0–6 m scales, but showed significant negative correlations at 6–10 m scales (Figure 4g). No significant spatial correlations were observed between snags of Moso bamboo and snags of other tree species across 0–10 m scales (Figure 4h).

During the late expansion stage of Moso bamboo, living stems and snags in MF showed positive correlations at 0–2 m scales, but these associations became non-significant beyond 2 m across 0–10 m scales (Figure 4i). Specifically, a positive correlation was observed between living and dead Moso bamboo stems at 0–2 m scales (Figure 4j), while no significant correlation existed between living stems and snags of other species (Figure 4k). Living Moso bamboo exhibited significant or near-significant positive correlations with living trees of other species at 0–2 m scales, with associations diminishing above this threshold (Figure 4l). Notably, snags of Moso bamboo showed no significant spatial correlations with snags of other tree species across all 0–10 m scales (Figure 4m).

## 3. Discussion

In CF, *Schima superba*, *Pinus massoniana*, and *Cunninghamia lanceolata* exhibited the highest abundance and basal area of snags (Table 1), a pattern directly linked to the dominance of their living counterparts in the stand. In contrast, *Castanopsis eyrei*, *Liquidambar formosana*, and *Cyclobalanopsis glauca* displayed significantly lower values in both snag metrics, which may reflect species–specific adaptations and divergent life-history strategies [28]. With Moso bamboo expansion, living bamboo stems gradually assumed dominance in TF, while living trees of other species mostly showed no significant overall changes in density and basal area, appearing minimally affected. However, the increased proportion of small-diameter saplings (5 ≤ DBH < 10 cm) likely resulted from mortality of mature trees and subsequent replacement by regenerating saplings of pioneer species. For example, over 60% of *Castanopsis eyrei* DBH < 10 cm. This inference was further corroborated by elevated total abundance and basal area of snags. Notably, bamboo expansion accelerated the mortality rate of native tree species in TF. The increase in the density of snags was also a manifestation of the rapid increase in bamboo density caused by the expansion of bamboo, which leads to the enhancement of intraspecific and interspecific competition within a short period of time [29]. During the late expansion phase, living and dead Moso bamboo stems established absolute dominance within the Moso bamboo-dominated forest. Increased diameter at breast height (DBH) of bamboo culms enhanced their capacity to capture light and nutrient resources, thereby accelerating their growth and expansion dynamics [30,31]. The resulting increased understory canopy closure critically suppressed natural regeneration of pioneer species, leading to near-extirpation of light-demanding species (*Cunninghamia lanceolata*, *Liquidambar formosana*, *Schima superba*) and the number of *Pinus massoniana* has also decreased, with only remnant large individuals persisting through their upper canopy. In contrast, shade-tolerant species (*Castanopsis eyrei*, *Cyclobalanopsis glauca*) demonstrated greater resistance, maintaining viable growth under bamboo-dominated conditions.

Stem diameter class distributions reflect growth status and mortality patterns [32], and analyzing structural shifts in living stems and snags provides critical insights into forest succession processes. In this study, distinct differences emerged in diameter class distributions between living stems and snags across the three stand types. Specifically, living stems in CF exhibited a complete diameter structure. Snags such as *Schima superba*, *Pinus massoniana*, and *Cunninghamia lanceolata* displayed diameter class distributional similarities to their living counterparts, showing comparable mortality rates between different diameter classes. This pattern aligns with Lorimer et al. [33], with a conclusion that the population size of living stems primarily governs deadwood abundance. The TF exhibited a marked increase in the proportion of living stems with DBH 10–15 cm during Moso bamboo expansion, primarily attributed to the substantial rise in bamboo density, as most Moso bamboo culms within these stands fall within this diameter range. Concurrently, the elevated proportion of living stems with DBH 5–10 cm likely resulted from canopy gaps created by mortality of original trees during bamboo invasion, facilitating recruitment of regenerating saplings and growth of small-diameter individuals. Notably, dead standing stems showed higher abundance in the small-diameter class (5 ≤ DBH < 10 cm), potentially reflecting greater vulnerability of these juvenile stems to growth constraints [34]. The relatively low mortality rate of DBH 10–15 cm individuals may derive from Moso bamboo’s intrinsic advantages over competing species, including rapid growth rates, high clonal propagation capacity, and phenotypic plasticity [35], which enhance resource acquisition efficiency and competitive exclusion of neighboring trees. However, as bamboo dominance intensified, mortality rates escalated among large-diameter trees, leading to their gradual exclusion from the community. Meanwhile, a significant mortality increase emerged in the DBH 10–15 cm class during late expansion phases, likely driven by intensified intraspecific competition following bamboo population saturation.

The spatial distribution of living stems and snags is jointly mediated by individual traits, interspecific interactions, and environmental drivers [36]. Our investigation documented and marked that the spatial pattern of living stems in the three stands changed significantly, suggesting that Moso bamboo expansion had a significant effect on the distribution pattern of living stems. These findings contrast with natural forest communities where plant species typically manifest aggregated distribution patterns [37]. Spatial point pattern analysis revealed that living stems in CF exhibited random distribution at finer scales (<5 m), transitioning to weakly aggregated patterns as spatial scales approached 10 m. This may be due to the high proportion of large-diameter living stems in CF, where a constant natural thinning process breaks up the aggregation early in the growth phase, so that living stems show a predominantly random distribution pattern. At larger scales, the aggregation may be due to habitat variability or patchiness that affects the spatial distribution of living stems [38]. When Moso bamboo expanded into CF, Moso bamboo living stems first showed a strong aggregation distribution, and then the aggregation intensity gradually weakened. This may be due to the fact that the distribution of Moso bamboo was more concentrated in the early stage of expansion under stems’ spatial constraints. In addition, the aggregated spatial pattern of Moso bamboo helps them to enhance clonal integration, which not only enhances stress tolerance, but also facilitates emergent community competitiveness and significantly improves survival rates [35]. The distribution patterns of other tree species show aggregation distribution at 0–4 m scales, transitioning to random distribution as the spatial scale increases. This pattern may result from bamboo expansion-induced mortality of large-diameter stems (DBH > 10 cm) in adjacent areas, which subsequently elevates the density of juvenile stems (5 ≤ DBH < 10 cm) around deadwood. During the growth of small-diameter living stems (DBH < 10 cm), intense competition arises from high niche overlap, leading to strong spatial aggregation of small-diameter deadwood. As the scale expands, limited seed dispersal capacity increasingly constrains propagule distribution, enhancing the probability of shifting from aggregated to random spatial patterns [32]. Furthermore, as the number of Moso bamboo increased, the Moso bamboo population’s demand for environmental resources intensified, experiencing intraspecific and interspecific competition and environmental screening, and a large number of Moso bamboo and the original trees in the forest died, eventually tending towards a random distribution [39]. The decrease in aggregation intensity favors the acquisition of sufficient environmental resources by Moso bamboo, and this change in aggregation intensity may be a survival strategy or adaptive mechanism for Moso bamboo expansion. The random distribution of snags at the 0–10 m scale in CF and MF suggests that the formation of snags in the pre- and post-expansion stages of Moso bamboo expansion is mostly random, and that natural senescence, successional elimination, and other spatially stochastic factors may be the main cause of tree mortality. In contrast, snags showed aggregated distribution at large scales in TF, suggesting that intra- and inter-species competition for survival space was the dominant factor in tree mortality during the middle stage of Moso bamboo expansion.

Positive and negative correlations between populations may be the result of both the effects of interspecific interactions and the convergence or divergence of species’ habitats [40]. In this study, there was no significant spatial correlation between living stems and snags in MF, which may suggest that tree mortality is not due to resource competition between individuals, and that natural mortality, environmental changes, pests and diseases dominate the causes. In TF, living stems and snags were significantly positively correlated, with the positive correlation between living trees of Moso bamboo and snags strengthened, while living stems of other species and snags showed no correlation at most scales. This suggests that there is strong competition between Moso bamboo and other tree species, Moso bamboo expansion contributes to the formation of snags, and Moso bamboo is more competitive for environmental resources. Meanwhile, the significant negative correlation between living stems of Moso bamboo and those of other larger diameter species (DBH > 10 cm) at larger scales also confirmed the strong competitive relationship between Moso bamboo and other tree species. While the positive correlation between Moso bamboo and young trees of other species (5 ≤ DBH < 10 cm) reached or approached a significant positive correlation at small scales, and a negative correlation at large scales. This indicates that the expansion of Moso bamboo promoted the growth of young trees to a certain extent during this stage. This is most likely because the expansion of Moso bamboo led to the death of large diameter trees around the Moso bamboo, and after the death of the trees, they created good nutrient conditions and spatial environments for the growth of young trees, and the density of new trees around the dead trees became larger. As the scale increases, the spatial correlation between Moso bamboo and other young trees gradually changes to a negative correlation, indicating that Moso bamboo inhibits other young trees at a larger scale, and it is difficult for these young trees to grow naturally around Moso bamboo. In contrast, within the MF, living stems and snags, and living stems of Moso bamboo and other tree species, only approached or reached a significant positive correlation at small scales, while at larger scales there was no significant correlation. It means that in the late stage of the expansion of Moso bamboo, the intra- and inter-species competition of Moso bamboo was weakened, and the inter-species correlation also changed from negative correlation in the middle stage of the expansion (TF) to positive correlation and non-correlation in the late stage (MF) [41]. The establishment of monodominant bamboo forests results in simplified community structure and reduced species diversity, exhibiting an inverted “J”-shaped diameter distribution pattern indicative of a stable community [42], which effectively impedes the natural succession of native coniferous and broadleaf mixed forests [43]. Therefore, we propose the following hypothesis: Moso bamboo drives irreversible succession from mixed coniferous–broadleaf forests to mono-dominant bamboo forests through an interspecific competition-mediated niche displacement mechanism. During the initial expansion phase (bamboo–tree mixed stage), Moso bamboo achieves competitive dominance via rhizome network expansion and rapid growth, suppressing large-diameter tree species (DBH > 10 cm) and triggering mortality hotspots (deadwood clusters). The vacated niches and nutrient resources from dead trees transiently promote regeneration of juvenile trees (5 ≤ DBH < 10 cm) at small scales. However, as expansion progresses, Moso bamboo establishes inhibitory effects at larger scales through underground resource monopolization and canopy shading, ultimately hindering sapling recruitment. In the late expansion phase (mono-specific bamboo forest stage), interspecific competition diminishes as dominance stabilizes. The clonal propagation of Phyllostachys edulis forms a homogenized community with an inverted-J diameter distribution, indicating self-sustaining regeneration that disrupts native forest succession. This process reflects a three-phase mechanism—competitive exclusion, resource reallocation, and community restructuring—whereby Moso bamboo replaces heterogeneous mixed forests with a mono-dominant, stable bamboo ecosystem, ultimately achieving complete ecological dominance.

## 4. Research Site and Methods

### 4.1. Research Site

**Figure 5:**
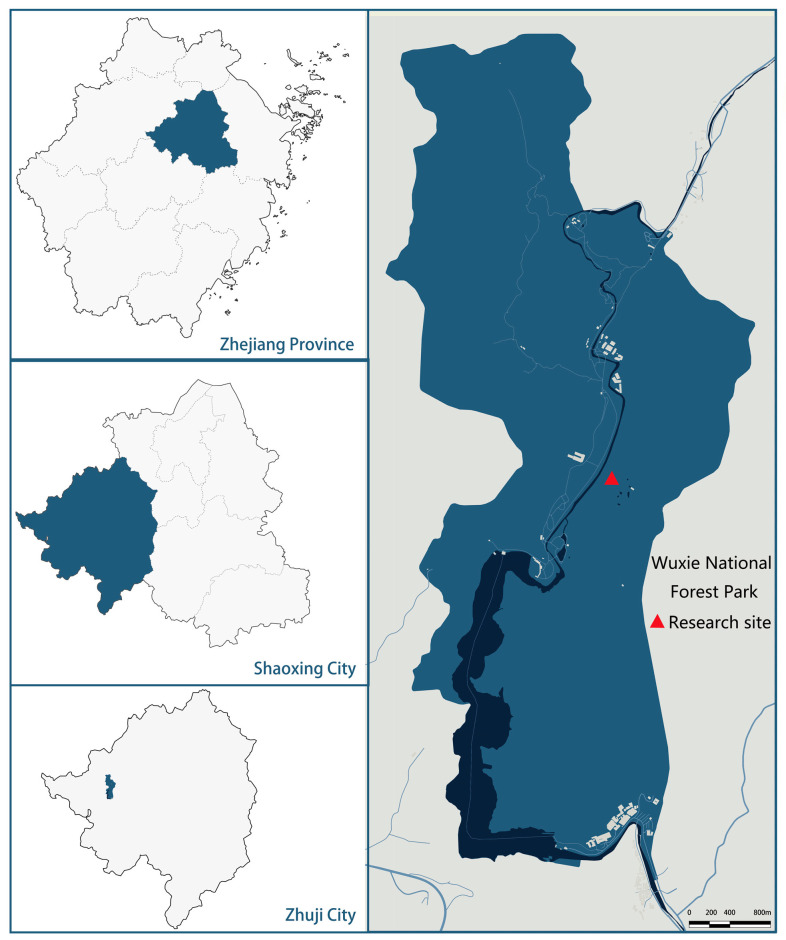
The location map of the study area.

The study area is situated within Zhejiang Wuxie National Forest Park (29°44′15″ N, 120°02′40″ E), geologically belonging to the northeast-trending branch of the Longmen Mountain Range (Figure 5). This region exhibits a characteristic subtropical monsoon humid climate (Cfa) with distinct seasonal variations, while the altitude is generally below 500 m, featuring a mean annual temperature of 16.2 °C and annual precipitation averaging 1347 mm. The dominant soil type is Ferralsols, and bedrock is mainly volcanic rocks and granite. The vegetation structure is dominated by coniferous-broadleaved mixed forests and Moso bamboo (*Phyllostachys edulis*) forests. Owing to the vigorous rhizome expansion capacity of Moso bamboo, a transitional forest belt—characterized as bamboo–arbor mixed forest—has naturally formed at the ecotone between these two primary vegetation types. The main plant species of coniferous broad-leaved mixed forest include *Pinus massoniana*, *Cunninghamia lanceolata*, *Schima superba*, *Liquidambar formosana*, *Castanopsis eyrei*, *Cyclobalanopsis glauca*, etc.

### 4.2. Experimental Design and Methodology

**Figure 6:**
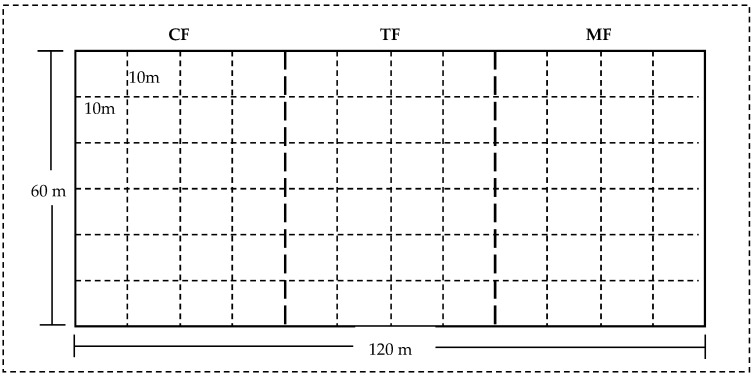
Schematic diagram of experimental plots.

Previous studies have demonstrated that sampling plot size significantly influences spatial pattern interpretation in point pattern analysis [44]. Notably, excessively large plots (>1 ha) may predominantly reflect spatial characteristics of habitat patches while masking individual-level patterns of plant populations. Conversely, insufficiently small plots (<0.04 ha) may fail to adequately capture population-level spatial distribution features due to limited sampling representativeness [45]. To investigate spatial pattern relationships between live and dead stems at 0–10 m scales across distinct bamboo invasion stages, a 120 m × 60 m permanent plot along Moso bamboo expansion front transitioning into coniferous and broad-leaved mixed forest (aligned with the width of the transitional forest belt) was established in 2023 on a gently sloping terrain within a subtropical transitional forest. The plot, surrounded by a 10 m buffer zone to mitigate edge effects, encompassed three forest types representing sequential bamboo (Phyllostachys edulis) expansion phases: (1) coniferous and broad-leaved mixed forest (CF, uninvaded by bamboo), (2) transition forest (TF, bamboo-to-tree basal area ratio ≈ 1:1), and (3) Moso bamboo forest (MF, near-monoculture stand), formed through bamboo natural colonization into coniferous–broadleaf forests, leading to progressive mortality of native tree species (Figure 6). A total of 72 10 m × 10 m quadrats (24 per forest type) were included (Table 2). All living stems with a diameter at breast height (DBH) ≥ 5 cm, including snags, were permanently tagged and georeferenced. Standardized measurements encompassed species identification, DBH (recorded to 0.1 cm precision), and spatial coordinates relative to quadrat origin (±10 cm positional accuracy).

### 4.3. Statistical Analysis

Spatial distribution patterns of standing stems were analyzed using Ripley’s L-function, an improved version of Ripley’s K-function [46]. The K-function quantifies spatial aggregation by evaluating the expected number of plants within radius r across the study area, defined as $K\left(r\right)=\frac{A}{n^{2}}\sum_{i=1}^{n}\;\sum_{j=1}^{n}\frac{1}{w_{ij}}I_{r}(u_{ij})(i\neq j)$, where *K(r)* is cumulative spatial clustering intensity at scale *r*. A is plot area, n is the individuals count within the sampling domain, *u_ij_* is the distance between two points *i* and *j*, *I_r_* (*u_ij_*) is the indicator function, (=1 if *u_ij_* ≤ *r*, else = 0), and *w_ij_* is the edge correction weight accounting for incomplete circular sampling at plot boundaries. Compared to *K(r)*, *L(r)* provides a more intuitive visualization of computational results in graphical representations. The calculation formula for Ripley’s L function is expressed as follows: $L\left(r\right)=\sqrt{k(r)/\pi }$ [47]. The 99% confidence envelope was established through the Monte Carlo simulation method. *L*(*r*) values exceeding the upper simulation envelope indicate a clustered distribution pattern, while those falling between the upper and lower envelopes suggest random distribution. Values below the lower envelope demonstrate uniform distribution characteristics. Spatial relationships were analyzed using bivariate point pattern analysis. The fundamental formula is $k_{12}\left(r\right)=\frac{A}{n_{1}n_{2}}\sum_{i=1}^{n}\;\sum_{i=1}^{n}\frac{1}{w_{ij}}I_{r}(u_{ij})(i\neq j)$ [48]. The square root transformation yielded $L_{12}\left(r\right)=\sqrt{\frac{K_{12}(r)}{\pi }}$. Spatial correlations between living and dead standing stems were investigated using the bivariate pairwise function *L*_12_(*r*), which simultaneously characterizes inter-group relationships and intra-group spatial patterns. A Monte Carlo simulation procedure generated 99% confidence envelopes. When *L*_12_(*r*) values fall within the simulation envelopes, spatial independence is indicated. Values exceeding the upper envelope demonstrate significant positive spatial association (attraction), whereas values below the lower envelope reveal negative spatial association (repulsion). All analyses were conducted using the spatstat package in R 4.1.3 [47].

Spatial distribution patterns inherently vary across scales. While the maximum spatial lag is conventionally set to half the length of the shortest side of rectangular plots in spatial analysis, this study adopted a more stringent threshold of one-quarter the shortest side length (i.e., maximum distance = 10 m) to minimize edge effects and sampling artifacts [49].

Twenty-four 10 m × 10 m quadrats per forest stand were established as sampling units. Stand type differences were analyzed through one-way ANOVA with post hoc LSD tests, maintaining a significance threshold (α) of 0.05. Relative abundance was calculated as the ratio of a species’ stem count to the total stem count within the stand. Relative dominance was defined as the proportion of a species’ total basal area relative to the cumulative basal area of all species. Data analyses and visualization were performed using SPSS (version 26.0) and RStudio (2022.02.0) statistical computing environments.

## 5. Conclusions

(1) Early Expansion Phase: The surrounding forest community (CF) exhibits high tree species diversity with unimodal diameter-class structures. Living stems and snags have random spatial patterns. Tree mortality is primarily driven by environmental stressors and senescence processes.
(2) Middle Expansion Phase: The forest community (TF) exhibits intensified competitive dynamics, characterized by species diversity decline, diameter-class structures simplification, tree mortality rates increase significantly, and clustered distribution of living stems/snags. Intra- and inter-species competition becomes the dominant cause of plant death.
(3) Late Expansion Phase: Moso bamboo establishes absolute dominance within the plant community, leading to weakened aggregating of both living stems and snags, and stabilized forest succession with negligible structural or spatial dynamics.
(4) Overall Impact: Moso bamboo expansion drives stand structural simplification through spatial decoupling of live stems and snags in adjacent forest stands across all stages.

## Figures and Tables

**Table 1:** Quantitative characteristics of living standing stems and snags.

	Objective Stems	Stand Types	Stem Species								
			*Phyllostachys* *edulis*	*Schima* *superba*	*Castanopsis* *eyrei*	*Pinus* *massoniana*	*Cyclobalanopsis* *glauca*	*Liquidambar* *formosana*	*Cunninghamia* *lanceolata*	Others	Total
Density/ (stem·ha^−1^)	living stems	CF	0 ^c^	100 ^a^	63 ^a^	50 ^a^	38 ^a^	33 ^b^	29 ^a^	46 ^a^	358 ^c^
		TF	1354 ^b^	83 ^a^	79 ^a^	63 ^a^	58 ^a^	71 ^a^	21 ^a^	46 ^a^	1775 ^b^
		MF	2438 ^a^	4 ^b^	75 ^a^	33 ^a^	42 ^a^	0 ^c^	0 ^b^	0 ^b^	2592 ^a^
	Snags	CF	0 ^c^	8 ^a^	4 ^a^	8 ^a^	4 ^a^	4 ^a^	8 ^a^	0 ^a^	38 ^c^
		TF	54 ^b^	17 ^a^	17 ^a^	17 ^a^	29 ^a^	4 ^a^	4 ^a^	0 ^a^	142 ^b^
		MF	396 ^a^	8 ^a^	13 ^a^	33 ^a^	13 ^a^	0 ^a^	8 ^a^	0 ^a^	471 ^a^
Basal area/ (m^2^·ha^−1^)	living stems	CF	0 ^c^	4.64 ^a^	2.50 ^a^	3.93 ^ab^	0.58 ^a^	2.11 ^a^	1.92 ^a^	1.12 ^a^	17.03 ^c^
		TF	15.36 ^b^	2.34 ^ab^	1.28 ^a^	7.37 ^a^	0.95 ^a^	2.68 ^a^	0.21 ^ab^	0.52 ^a^	30.72 ^b^
		MF	34.03 ^a^	0.08 ^b^	2.74 ^a^	2.34 ^b^	1.02 ^a^	0 ^b^	0 ^b^	0 ^b^	40.48 ^a^
	Snags	CF	0 ^c^	0.26 ^a^	0.06 ^a^	0.55 ^a^	0.08 ^a^	0.03 ^a^	0.58 ^a^	0 ^a^	1.55 ^b^
		TF	0.47 ^b^	0.20 ^a^	0.17 ^a^	1.31 ^a^	0.27 ^a^	0.04 ^a^	0.26 ^a^	0 ^a^	2.71 ^b^
		MF	5.12 ^a^	0.08 ^a^	0.26 ^a^	2.84 ^a^	0.27 ^a^	0 ^a^	0.78 ^a^	0 ^a^	9.36 ^a^

The differences in density and basal area of living stems and snags among the three forest stands were analyzed using one-way ANOVA with post hoc LSD tests. Significant differences (*p* < 0.05) exist among different forest types indicated by the letters in the same column.

**Table 2:** General condition of experimental plots.

Stand Types	Average Altitude	Slop Aspect	Slop Degree	Average Diameter	Dominant Families	Dominant Species	Life Form
CF	228 m	WN	8°	22.9 cm	*Fagaceae*	*Schima superba*	Tree
					*Theaceae*	*Castanopsis eyrei*	Tree
					*Pinaceae*	*Pinus massoniana*	Tree
					*Altingiaceae*	*Cyclobalanopsis glauca*	Tree
					*Cupressaceae*	*Liquidambar formosana*	Tree
TF	236 m	WN	14°	13.38 cm	*Poaceae*	*Phyllostachys edulis*	Grass
					*Fagaceae*	*Schima superba*	Tree
					*Theaceae*	*Castanopsis eyrei*	Tree
					*Altingiaceae*	*Liquidambar formosana*	Tree
					*Pinaceae*	*Pinus massoniana*	Tree
MF	242 m	WN	15°	13.78 cm	*Poaceae* *Fagaceae* *Pinaceae*	*Phyllostachys edulis*	Grass
						*Castanopsis eyrei*	Tree
						*Cyclobalanopsis glauca*	Tree
						*Pinus massoniana*	Tree

## Data Availability

The data presented in this study are available on request from the corresponding author.
